# Burnout, Attachment and Mentalization in Nursing Students and Nurse Professionals

**DOI:** 10.3390/healthcare9111576

**Published:** 2021-11-18

**Authors:** Giulia Bordoagni, Edita Fino, Alessandro Agostini

**Affiliations:** 1 Infermi Hospital of Rimini, 47923 Rimini, Italy; giulibordo96@gmail.com; 2 Department of Experimental, Diagnostic, and Specialty Medicine (DIMES), S. Orsola-Malpighi Hospital, Alma Mater University of Bologna, 40138 Bologna, Italy; alessandro.agostin11@unibo.it

**Keywords:** nursing students, professional nurses, attachment style, mentalization, burnout

## Abstract

(1) Background. In caretaking professions, attachment style and mentalization capacities are essential factors for establishing an effective caretaker–patient relationship and for buffering burnout. While attachment avoidance and dependency are considered risk factors for burnout, impairment in mentalization capacity is associated with psychological distress and ineffective emotion regulation. (2) Objective: Evaluating the attachment style and mentalization capacity in nurse professionals and nursing students. We further investigated the impact of these factors on burnout in professional nurses. (3) Method: 94 nursing students and 94 controls and 34 professional nurses completed the Attachment Style Questionnaire (ASQ) and the Reflective Functioning Questionnaire (RFQ). For professional nurses, the Maslach’s Burnout Inventory (MBI) was also administered. (4) Results: Nursing students exhibited lower scores in secure attachment and higher scores in anxiety over relationships compared to controls while no difference in mentalization capacity was found between both groups. Importantly, attachment anxiety resulted a significant predictor of burnout in professional nurses. (5) Conclusions: Nursing students might compensate their attachment insecurity with high mentalization. Attachment security may play a protective role against burnout in the professional nurses. Education programs aimed at enhancing mentalizing abilities might facilitate nursing students’ entrance in the forthcoming clinical environment and practice. Implementing training strategies based on attachment theory may contribute to burnout prevention in nurse professionals.

## 1. Introduction

The ability to establish positive and trusting therapeutic relationships with patients is widely recognized as an essential component of nursing practice and effective delivery of care [1]. Among other factors, attachment style and mentalizing capacities have emerged as salient individual factors involved in the development of the therapeutic caretaking relationship [2,3]. Attachment style refers to a characteristic orientation to the other person in an interpersonal relationship that originates in early relationships with parents or other key figures and is broadly categorized as secure or insecure [4]. A secure attachment develops if caregivers are experienced as reliably available and responsive to one’s needs and is characterized by an inner sense of safety and effective regulation of affects. In contrast, when caregivers are experienced as unavailable or inconsistently responsive an insecure attachment style develops which is characterized by two fundamental dimensions labeled as anxiety over relationships and avoidance or discomfort with closeness [4,5,6,7]. Early infant–caregiver attachment relationships serve as a foundation for the development of other critical capacities such as mentalization, which denotes the ability to think about one’s own emotions and desires, as well as to empathize and reflect on feelings and needs of others [8]. Otherwise known by the term reflective functioning, mentalization capacities mature over the course of development through sustained interpersonal relationships and are crucial for caregiving relationships. Indeed, research shows that securely attached caregivers may be better able to appropriately respond to patients with emotional distress compared to the insecurely attached caregivers [1,2,3]. Attachment insecurity on the other hand, combined with low empathy or mentalization capacity are associated with burnout, the syndrome of physical and emotional exhaustion, depersonalization, and inefficiency at work which plagues caretaking professions such as nursing [9].

Among healthcare professions, nursing is perhaps the most demanding as it requires delivering compassionate care in often stressful and adverse conditions and to effectively navigate complex interpersonal relations with patients, families and other healthcare professionals [10,11]. If more insecurely attached individuals cope less well with stress, are tendentially ill-equipped in mentalization capacities that facilitate interpersonal relationships and are more prone to burnout, then adult attachment insecurities and poor mentalization capacity may be expected to predict burnout in nurse professionals. While the mechanisms through which attachment style and mentalization capacity affect burnout in caretaking professionals need to be more thoroughly elucidated, one study found that mentalization, but not attachment style predicted the effectiveness of psychotherapists and that high mentalization ability compensated for insecure attachment [12]. To our knowledge, no study has yet examined the relationship of attachment insecurity, mentalization capacity and burnout in the nursing profession.

The purpose of the present study was to address this issue and investigate the interrelationship between attachment style, mentalization and burnout in the nursing workforce. The objective was that of evaluating attachment style and mentalization capacity in nursing students as important individual factors in their chosen line of work. Second, we aimed to shed light on the ways that attachment style and mentalizing capacity may affect burnout in professional nurses. 

## 2. Materials and Methods

### 2.1. Study Design and Sampling

A case–control cross-sectional design was used to assess attachment style and mentalization in nursing students and controls and the impact of these factors on burnout was further assessed in a sample of professional nurses. All third-year nursing students attending the Nursing School of University of Bologna (courses of Faenza and Rimini) were invited to participate in the study. Recruitment of participants took place over a three-month period via information meetings held after lecture during which participants were verbally explained the purpose of the study and that participation was on voluntary basis. The inclusion criteria were being a nursing student and a native Italian speaker. For the recruitment of the control group, advertisements and posters were exposed in various university premises and supermarkets in Bologna. Inclusion criteria were being 18 years or older, whereas exclusion criteria involved being a health care provider (i.e., nurse, medical doctor, psychotherapist, medical student, nursing student) and non-native Italian speaker.

To recruit the group of nurse professionals, an invitation email was sent to all nurses of the S. Orsola-Malpighi University Hospital of Bologna to participate in a voluntary paper-based survey. The recruitment process took place over a three-month period and inclusion criteria were being a registered nurse directly delivering care in various clinical units, with at least one year of professional experience, and being a native Italian speaker. The study was conducted at the University of Bologna and trained psychologists of the Department of Experimental, Diagnostic, and Specialty Medicine (DIMES) administered the psychometric questionnaires. The study was approved by the Institutional Review Board of University of Bologna (Protocol Nr: 13264, date 15 January 2018), and all participants provided written consent. 

### 2.2. Data Collection Instruments

To investigate the attachment style, we used the Attachment Style Questionnaire (ASQ) [13] which is a 40-item self-report questionnaire designed to assess the attachment dimensions. ASQ items consist in statement such as “I think it is important that people can rely on each other” and participants are asked to rate their level of agreement or disagreement on a 6-points Likert scale, where 1 = “totally disagree” and 6 = “totally agree”. The ASQ contains 5 subscales labelled as: (1) confidence (8 items), describing secure attachment, (2) discomfort with closeness (10 items) and (3) relationships as secondary (7 items), both assessing attachment avoidance, (4) need for approval (7 items) and (5) preoccupation with relationships (8 items), both describing attachment anxiety. 

Mentalizing capacity was measured with the brief version of the Reflective Functioning Questionnaire, (RFQ) [14] which is a self-report questionnaire composed of eight items such as “Sometimes I do things without really knowing why”. Items are scored on a 7-point Likert scale, ranging from 1 = “completely disagree” to 7 = “completely agree”. The RFQ contains two subscales labeled as certainty (RFQc) and uncertainty (RFQu) about mental states. Low scores in RFQc describe people characterized by rigid certainty about the mental states they attribute to themselves and others (low mentalization), while high scores designate people who typically engage in thinking about how mental states influence behaviors (high mentalization). Conversely, high scores on RFQu designate people with difficulties in mentalization while low scores in RFQu reflect “the acknowledgment of the opaqueness of one’s own mental states and that of others, typical of genuine mentalizing”.

To measure burnout levels of nurse professionals we used Maslach’s Burnout Inventory (MBI) [15] a widely used questionnaire of 22 items which load on 3 factors: Emotional exhaustion (EE), measuring feelings of being emotionally drained in one’s work; Depersonalization (DP), measuring insensitivity and impersonal responses towards recipients of one’s care; and Personal achievement (PA), measuring feelings of being competent and successful in one’s work. Items are scored on a 7-point Likert scale in which 0 = “never” and 6 = “every day”. Based on research on nursing population a cut-off point >26 was considered as indicative of high level of emotional exhaustion, a cut-off score >9 was considered as indicative of severe depersonalization, and a cut-off point <34 as indicating severe deficiency in personal achievement.

### 2.3. Statistical Analysis

Statistical analyses were conducted using Statistical Package for Social Sciences (SPSS) software version 24 (IBM, Armonk, NY, USA). For group comparisons Chi-squared tests and analysis of variance (ANOVA, New Providence, NJ, USA) were performed. To assess the relationship between attachment dimensions and mentalizing variables Pearson’s r coefficients were computed for each group separately. To assess the role of Attachment style and Mentalizing on Burnout in professional nurses, we performed regression models using the stepwise method. Three separate multiple linear regression analyses were conducted to identify the role of demographic, attachment dimensions and mentalization measures on the three MBI subscales. Ten variables were entered into the model: age (in years), sex (being female), years of work, ASQ Confidence, ASQ Discomfort with closeness, ASQ Secondary with relationships, ASQ Need for approval, ASQ Preoccupation with relationships, RFQc and RFQu.

## 3. Results

### 3.1. Sample Characteristics

A total of 94 nursing students (71 females (75.5%) mean age 22.43 ± 2.62) and 34 professional nurses (32 females, 94.2%, mean age 47.21 ± 7.69) completed the study. The control group consisted of 94 subjects (68 females, 72.3% mean age 23.21 ± 5.29) matched with the nursing students’ group for sex, age and education. Table 1 shows the sociodemographic data and scores on psychometric measures for professional nurses, nursing students and the control group separately.

There were no differences in terms of sociodemographic variables of age, sex and education between nursing students and the control group. Compared to controls, nursing students exhibited lower scores in the ASQ Confidence dimension describing secure attachment (F = 4.44, *p* = 0.036) and higher scores in the ASQ Preoccupation with relationships dimension describing anxiety over relationships (F = 4.05, *p* = 0.045) (see Table 1). On the other hand, no group differences emerged in the comparison between the mentalization measures.

### 3.2. Assessing the Relationship between Attachment Style and Mentalization in Nursing Students

To assess the relationship between measures of attachment style (ASQ) and mentalization (RFQ) in nurse professional, nursing students and controls we computed Pearson’s coefficients separately for each group. In nursing students, the dimension ASQ preoccupation with relationships was negatively correlated with RFQ certainty (r = −0.26, *p* = 0.012) and positively correlated with RFQ uncertainty (r = 0.22, *p* = 0.03). In the control group, there was a positive correlation between ASQ preoccupation with relationships and RFQ certainty (r = 0.22, *p* = 0.027).

### 3.3. Assessing the Role of Attachment Style and Mentalization on Burnout in Nurse Professionals

For the nurse professional group, results on the burnout subscales of emotional exhaustion, depersonalization and personal achievement revealed mean scores above the indicated cutoff values suggested for clinical relevance of symptoms (see Table 1). No effects of age and professional seniority was found on burnout dimensions.

To further assess the relationship between attachment style and mentalization capacity on burnout in professional nurses a series of linear regressions were performed. In the first regression analysis with emotional exhaustion (EE) as dependent variable, there was a significant linear relationship between EE and the set of predictor variables: R^2^ = 0.15, F = 5.78 and *p* = 0.02. Among the parameters entered in the model, the significant predictor of EE was ASQ Need for approval (β = 0.39, *t* = 2.41, *p* < 0.022) (see Table 2). In the second regression analysis with depersonalization (DP) as dependent variable, the linear relationship between DP and the set of predictor variables was also significant: R^2^ = 0.23, F = 9.57, *p* = 0.004. Among the variables entered in the model, the significant predictor of DP was ASQ Need for approval (β = 0.48, *t* = 3.09, *p* < 0.004) (see Table 2). In the third regression analysis, the linear relationship between personal achievement (PA) and the set of predictor variables was R^2^ = 0.25, F = 11.08, *p* = 0.002. Like previous results, the significant predictor of PA was ASQ Need for approval (β = −0.51, *t* = −3.33, *p* = 0.002) (see Table 2).

## 4. Discussion

In the present study, two crucial dimensions for establishing positive and trusting caretaker–patient relationships, attachment style and mentalizing abilities were assessed across groups of nurse professionals, nursing students and controls. Our findings showed that compared to controls, nursing students exhibited higher scores in the dimensions revealing attachment insecurity, while no deficits emerged in terms of mentalization capacity. The role of attachment and mentalization on predicting burnout dimensions was further assessed in professional nurses with need for approval, one of the two main dimensions revealing attachment anxiety, resulting a significant predictor. 

In line with previous studies [1,2,3], the more pronounced insecure attachment exhibited by nursing students may affect their forthcoming clinical practice both in terms of their role as caregivers and as members of interdisciplinary healthcare teams. It may also represent a potential disturbing factor in the development of effective relationships with patients and families [11]. However, these findings should be discussed in the light of the results obtained on the mentalization measure. Mentalization allows the understanding of the needs of others and promotes attachment security facilitating effective interpersonal interactions and the development of trusting and positive relationships [8]. In the healthcare context, previous studies have found that therapists’ mentalization capacity influences their therapy effectiveness independently of attachment style [12], highlighting the role of high mentalization capacity in compensating for insecure attachment. Thus, it is reasonable to assume that nursing students may use the same mechanisms to compensate for their attachment insecurity. This has implications for nursing practice considering that mentalization abilities can be improved, both in patients with personality disorders and in healthy subjects though mentalization-based psychotherapies and training programs [8]. 

Our results importantly highlight the usefulness of the assessment of attachment style and mentalization in professional nurses to elucidate and reinforce the role of protective factors against burnout. While previous research has shown that attachment avoidance and dependency predict burnout, our findings extend this knowledge to suggest that attachment anxiety may contribute to increasing levels of burnout in professional nurses. Indeed, the need for interpersonal approval in nursing clinical settings may be associated with feelings of incompetence and lack of personal accomplishment promoting emotional distress and subsequent burnout. Although previous studies have found a negative association between burnout and empathy in nurse professionals [9], in the present study, mentalization (or lack thereof), a construct that encompasses empathy, was not a predictor of burnout. It may be that the use of a self-report measure of mentalization capacity such as the RFQ might have been affected by potential low self-awareness, and thus may have obscured our results.

The present study is not exempt from limitations. For instance, the low number of participants in the nurse professionals’ group and the single-centered nature of the study does not allow for generalization of results. Work-related factors such as working in different units (emergency, oncology, critical care, etc.) involving various degrees of exposure to interpersonal stress may have an impact on the roles and experience of participants and we did not directly address this issue. Future research involving a larger sample of nurse professionals and also including contextual variables such as workload or stressful life events are needed to more thoroughly investigate the interplay of these factors with attachment style, mentalization capacity and burnout in the nursing profession. 

## 5. Conclusions

In conclusion, results of the present study support the hypothesis that attachment insecurity may be a risk factor for burnout in professional nurses. In addition, the more pronounced attachment insecurity that we found in nursing students compared to controls suggests the usefulness of assessing attachment dimensions and mentalization capacity in both nursing students and nurse professionals with a view to developing and implementing undergraduate and on-job skill enhancement programs in these specific areas. Training programs aimed at enhancing reflective functioning skills might facilitate nursing students’ entrance in the forthcoming clinical environment and practice and contribute to professional nurses’ establishing more effective caretaking relationships.

## Figures and Tables

**Table 1:** Socio-demographic data and mean (±SD) scores obtained on psychometric scales are reported separately for the professional nurses, nursing students and control group.

Measured Variables	Professional Nurses (*n* = 34)	Nursing Students (*n* = 94)	Controls (*n* = 94)
Socio demographics			
Age (years)	47.21 (±7.69)	22.43 (±2.62)	23.21 (±5.29)
Sex (Male/Female)	2/32	23/71	26/68
Professional seniority	11.03 (±9.18)		
Psychometric measures			
Attachment Style			
ASQ Confidence	33.12 (±6.72)	30.36 (±4.43)	31.71 (±4.33)
ASQ Discomfort with closeness	35 (±7.29)	34.22 (±5.05)	32.99 (±4.63)
ASQ Relationships as secondary	15.5 (±6.13)	14.98 (±5.16)	14.85 (±4.36)
ASQ Need for approval	20 (±4.25)	21.95 (±5.26)	21.26 (±4.77)
ASQ Preoccupation with relationships	26.52 (±7.21)	30.22 (±6.50)	28.47 (±5.38)
Mentalization			
RFQ certainty	1.39 (±0.74)	1.02 (±0.62)	1.04 (±0.58)
RFQ uncertainty	0.52 (±0.41)	0.61 (±0.51)	0.65 (±0.52)
Burnout			
MBI Emotional exhaustion	23.82 (±10.62)		
MBI Depersonalization	4.32 (±4.08)		
MBI Personal achievement	39.64 (±6.33)		

Note: MBI = Maslach burnout inventory; ASQ = attachment style questionnaire; RFQ = reflective functioning questionnaire.

**Table 2:** Predictors of burnout in nurses (*n* =34).

Regression Model	MBI Emotional Exhaustion		MBI Depersonalization		MBI Personal Achievement	
Model Statistics						
*R* ^2^	0.15		0.23		0.26	
*F*	5.79 *		9.57 **		11.08 **	
Variables entered in the model	*β*	95% Cl	*β*	95% Cl	*β*	95% Cl
ASQ Need for approval	0.39 *	(0.15, 1.80)	0.48 **	(0.15, 0.76)	−0.51 **	(−1.21, −0.29)

* *p* < 0.05. ** *p* < 0.01. Cl = confidential interval; ASQ = attachment style questionnaire; RFQ = reflective functioning questionnaire; Predictor variables in the model included: age, sex, years of work, ASQ Confidence, ASQ Discomfort with closeness, ASQ Secondary with relationships, ASQ Need for approval, ASQ Preoccupation with relationships, RFQ certainty, RFQ uncertainty.

## Data Availability

Data supporting this article can be made available by the corresponding author upon reasonable request.
